# Artificial Intelligence Education and Tools for Medical and Health Informatics Students: Systematic Review

**DOI:** 10.2196/19285

**Published:** 2020-06-30

**Authors:** A Hasan Sapci, H Aylin Sapci

**Affiliations:** 1 Adelphi University Garden City, NY United States

**Keywords:** artificial intelligence, education, machine learning, deep learning, medical education, health informatics, systematic review

## Abstract

**Background:**

The use of artificial intelligence (AI) in medicine will generate numerous application possibilities to improve patient care, provide real-time data analytics, and enable continuous patient monitoring. Clinicians and health informaticians should become familiar with machine learning and deep learning. Additionally, they should have a strong background in data analytics and data visualization to use, evaluate, and develop AI applications in clinical practice.

**Objective:**

The main objective of this study was to evaluate the current state of AI training and the use of AI tools to enhance the learning experience.

**Methods:**

A comprehensive systematic review was conducted to analyze the use of AI in medical and health informatics education, and to evaluate existing AI training practices. PRISMA-P (Preferred Reporting Items for Systematic Reviews and Meta-Analysis Protocols) guidelines were followed. The studies that focused on the use of AI tools to enhance medical education and the studies that investigated teaching AI as a new competency were categorized separately to evaluate recent developments.

**Results:**

This systematic review revealed that recent publications recommend the integration of AI training into medical and health informatics curricula.

**Conclusions:**

To the best of our knowledge, this is the first systematic review exploring the current state of AI education in both medicine and health informatics. Since AI curricula have not been standardized and competencies have not been determined, a framework for specialized AI training in medical and health informatics education is proposed.

## Introduction

### Overview

Artificial intelligence (AI) is one of the most disruptive innovations in health care, and the topic has attracted the attention of physicians, clinicians, researchers, and medical device industry professionals. Recent advancements in machine learning (ML) and deep learning (DL) algorithms and cloud computing have increased the adoption of AI. Consequently, applications that can handle a large number of unstructured data sets and solve complex problems have become a part of daily clinical practice.

Most AI applications process data and run self-learning algorithms behind the scenes. Although some AI applications provide data-driven recommendations to clinicians, others may not offer an option to accept, reject, or modify the output. The recommendations AI applications provide through statistical correlations may not be the best option because human-made AI algorithms may be flawed. To use and screen AI-based decisions, clinicians and health informaticians who develop AI applications should have an excellent understanding of the underlying AI concepts. This paper will focus on the emerging need for formal AI education in medicine and health informatics.

### Background

Intelligence requires the capacity to perceive contexts, associate contexts to actions, and act. Even though the concept of machines that imitate intelligent human behavior is not new, AI has recently become a topic of interest [1]. As an academic discipline, the Dartmouth College Artificial Intelligence Conference that was organized by John McCarthy in 1956 was considered the birth of this field [2].

AI, ML, and DL are closely related, and the absence of universal definitions might be confusing; however, the difference between AI, ML, and DL is simple. AI is defined as “the theory and development of computer systems able to perform tasks normally requiring human intelligence, such as visual perception, speech recognition, decision-making, and translation between languages” [3]. AI-based devices can perceive the environment, simulate human intelligence, and solve problems. Their ability to adapt through progressive learning algorithms is what differentiates AI technologies from robotic and hardware-driven automation. In other words, computers can mimic human intelligence using AI techniques [4]. ML is the subset of AI that allows systems to learn from data and develop self-learning algorithms. ML applications can learn from data without being explicitly programmed; make predictions and recommendations using various tools; and enable computer applications to improve their performance [5]. DL is a subfield within ML that allows machines to use algorithms inspired by the structure of neural networks. A computer can learn how to classify images and how to assign labels to words in a sentence (semantic labeling) by using DL algorithms [5]. DL programming uses large quantities of unstructured data, calculates complex statistical models, and predicts outcomes without being explicitly programmed. Virtual assistants, chatbots, and facial recognition algorithms are some other practical examples of DL.

**Figure 1:**
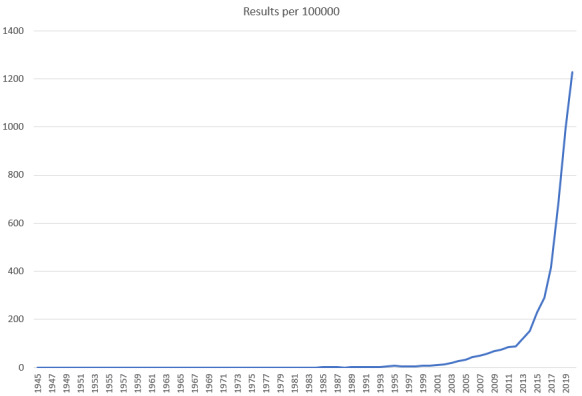
The number of citations that appear on PubMed by year for the following search terms: ("Medical Education" OR "Medical Training") AND ("Artificial Intelligence" OR "Machine Learning" OR "Deep Learning").

The main purpose of this study was to investigate peer-reviewed publications focused on AI education and to determine objective assessment methods for AI skills and competency training for medical and health informatics professionals. The impact of AI on the learning experience was evaluated to assess the need for AI education. As medical education, clinical informatics education, and health informatics education are closely related, medical and health informatics education trends were analyzed together. The American Medical Informatics Association has been working closely with the Commission on Accreditation for Health Informatics and Information Management Education to determine health informatics competencies; clinical informatics became a medical subspecialty in 2011 [6]. Although clinical and health informatics programs are designed for students who plan to pursue different career pathways, they use similar competencies. The implementation of ML in health care could result in unintended challenges and biased decisions, depending on the algorithms, data sources, and methodologies used [7]. Physicians who are not familiar with the evidence standards for AI might not be able to use the right approaches to integrate AI into clinical care. Even though there are several studies that explored how AI algorithms were helping enhance education [8-12], the number of peer-reviewed publications that focused on artificial intelligence education in medicine is limited (Figure 1).

## Methods

Using a replicable systematic search strategy, a full-text review was performed between November 2019 and February 2020. PRISMA-P (Preferred Reporting Items for Systematic Reviews and Meta-Analysis Protocols) systematic review methodology introduced by Moher et al [13] was used to identify and analyze reliable literature. The PRISMA-P method uses a structured procedure that consists of a 17-item checklist to facilitate systematic review protocols.

**Figure 2:**
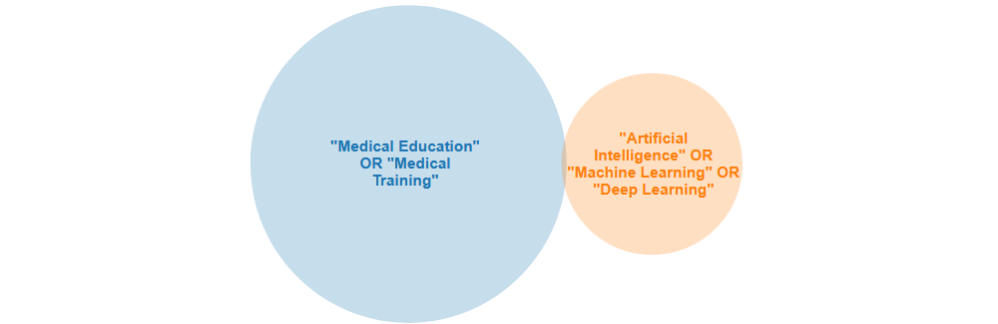
Venn diagram for the PubMed search. A total of 363 results were found with the following search terms: ("Medical Education" OR "Medical Training") AND ("Artificial Intelligence" OR "Machine Learning" OR "Deep Learning").

The combination of five groups of keywords was used to search PubMed, IEEE (Institute of Electrical and Electronics Engineers) Xplore Digital Library, CINAHL (Cumulative Index to Nursing and Allied Health Literature) Plus, and ScienceDirect databases: (1) medical education, (2) medical training, (3) artificial intelligence, (4) machine learning, and (5) deep learning (Figure 2 and Table 1). Overall, 2082 articles matched the search criteria. After removing duplicate studies and performing an abstract review, 76 full-text articles were selected for the review. All search results were entered into EPPI-Reviewer 4 text mining software (the EPPI-Centre, University of London), and the studies that met the inclusion criteria were identified. Two researchers performed the extraction independently and assessed quality.

**Table 1:** Literature sources and keywords.

Search query and literature sources		Search in		Return value
**(“Medical Education” OR “Medical Training”) AND (“Artificial Intelligence” OR “Machine Learning” OR “Deep Learning”)**				
	PubMed		All fields	363
	IEEE (Institute of Electrical and Electronics Engineers) Xplore		Full text and metadata	60
	ProQuest Central		Full text and peer reviewed	6271
	CINAHL (Cumulative Index to Nursing and Allied Health Literature) Plus		All text (TX)	68
	ScienceDirect		Title, abstract, author-specified keywords	1588

**Figure 3:**
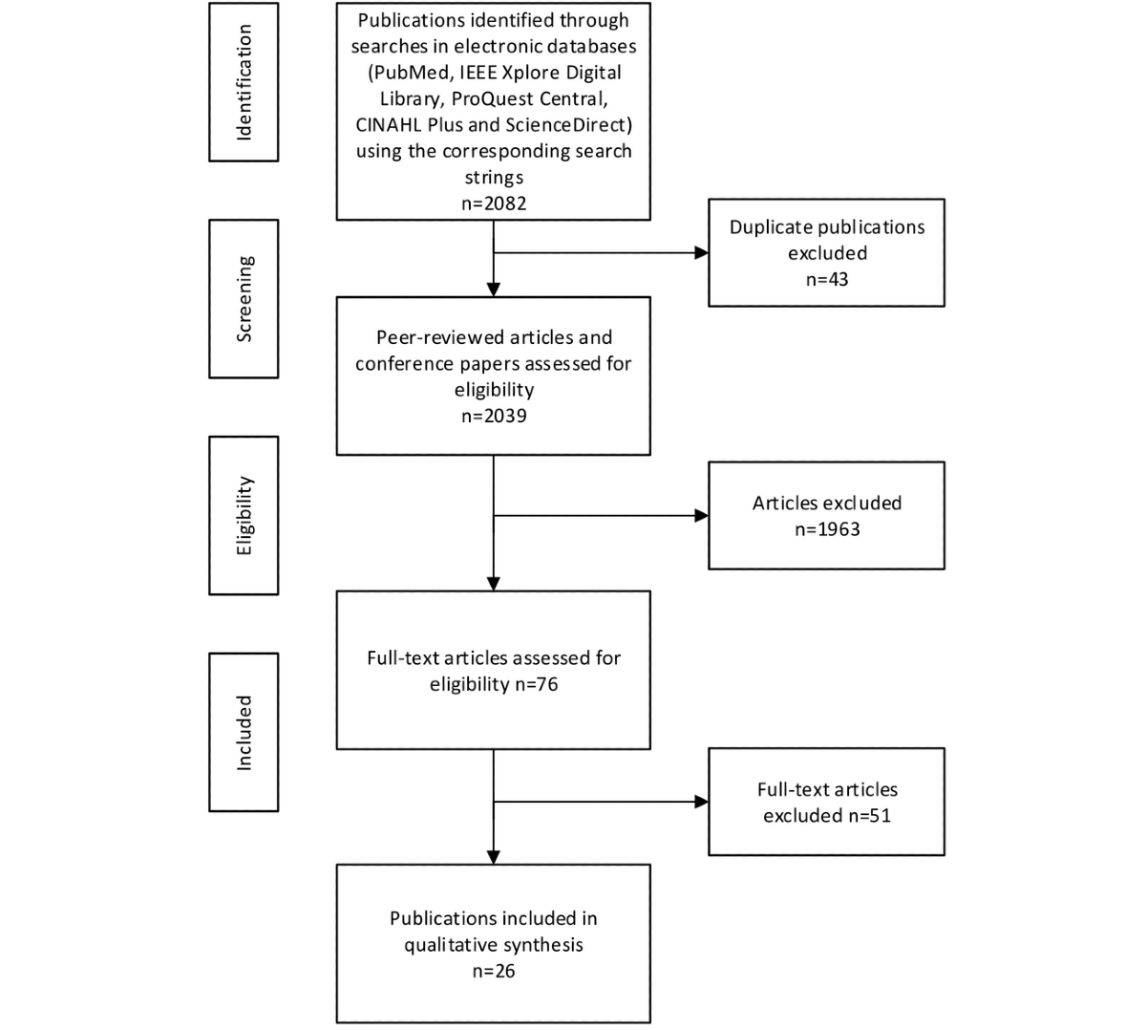
Search methodology. CINAHL: Cumulative Index to Nursing and Allied Health Literature; IEEE: Institute of Electrical and Electronics Engineers.

Based on the following inclusion and exclusion criteria, the selection process was applied. Peer-reviewed research articles, review papers, conference papers, case reports, correspondences, discussions, viewpoint papers, editorials, mini-reviews, and short communications papers that focused on AI tools to enhance the learning experience in medical and health informatics education or teach AI as a new competency published after 1990 were included (Table 1). Book chapters, news, and extended abstracts published before 1990 in languages other than English were excluded. To establish validity, disagreements were discussed until a consensus was reached. Overall, 26 articles matched the inclusion criteria of this research (Figure 3).

## Results

One of the goals of this systematic literature review was to evaluate existing studies and determine the current state of AI education. The selected papers were used to identify the answers to three research questions.

The first research question is the following: what topics are discussed in peer-reviewed publications that focus on medical and health informatics education and AI? To answer this question, the selected publications were classified based on their education foci and the characteristics of included studies were summarized in Table 2. The publications that focused on the use of AI applications in medical and health informatics education were categorized as Category 1. These studies used various AI-based tools to enhance the learning experience. Several case studies and new initiatives to teach specific AI skills, such as ML programming languages and big data analytics software, were also identified. The publications that evaluated AI education were classified as Category 2. Category 1 studies discussed different AI applications to enhance education and summarized the impact of AI on medical and health informatics education, while Category 2 studies focused on the teaching of AI concepts.

**Table 2:** Characteristics of included studies.

Author(s), year, and reference	Country	Title or objective	Category^a^	Level of evidence^b^	Study objective	Comments and knowledge gap
Winkler-Schwartz et al, 2019 [14]	Canada	Artificial Intelligence in Medical Education: Best Practices Using Machine Learning to Assess Surgical Expertise in Virtual Reality Simulation	1	IV	The authors developed a checklist to assess surgical expertise in virtual reality simulation.	The study provided a general framework only. The authors emphasized the need to add further elements.
Chan and Zary, 2019 [15]	Singapore	Applications and Challenges of Implementing Artificial Intelligence in Medical Education: Integrative Review	1	IV	This review evaluated current applications of AI^c^ in medical education and highlighted the main challenges.	The authors acknowledged that a low number of studies were reviewed and stated that conclusions might be inconsequential.
Lillehaug and Lajoie, 1998 [16]	Sweden	AI in medical education—another grand challenge for medical informatics	1	IV	This comprehensive review discussed the potential use of AI to enhance medical informatics education.	This article was published before the discovery of high-performance computing processors and recent advancements in data recording technology.
Frize and Frasson, 2000 [17]	Canada	Decision-support and intelligent tutoring systems in medical education	1	V	This study evaluated the use of intelligent tutoring systems in medical education.	This article discusses the potential use of decision support tools but emphasizes the need for further research to validate their usefulness.
Zhao et al, 2018 [18]	China	Research on Application of Artificial Intelligence in Medical Education	1	V	This article analyzed the application of AI in medical education.	This study evaluated the effect of AI technology on traditional medical education with a focus on personalized learning.
Chary et al, 2018 [19]	United States	A Review of Natural Language Processing in Medical Education	1	IV	This study reviewed the application of NLP^d^ to medical education and identified concepts from NLP used in those applications.	The authors investigated the integration of NLP to medical education resources using published manuscripts and stated the potentially biased representation of the scope.
Caudell et al, 2003 [20]	United States	Virtual patient simulator for distributed collaborative medical education	1	IV	The study investigated the feasibility of using a real-time AI simulation engine in medical school curricula.	The study described an ongoing project and did not provide any data about the difference between problem-based learning using virtual patient simulators and standard paper case tutorials.
Guimarães et al, 2017 [21]	Portugal	Rethinking Anatomy: How to Overcome Challenges of Medical Education's Evolution	1	IV	This literature review evaluated the integration of complementary technology-based methodologies to medical instruction.	The authors discussed the potential of AI in learning analytics-oriented systems to predict behavior but did not make any recommendations about new research studies.
Bowyer et al, 2008 [22]	United States	Immersive Virtual Environments for Medical Training	1	IV	This study highlighted the role of advanced virtual environments and surgical simulators as a training platform for medical training.	The paper described various virtual reality environments where students can interact with AI-based simulators.
Sitterding et al, 2019 [23]	United States	Using Artificial Intelligence and Gaming to Improve New Nurse Transition	1	IV	This research discussed the preliminary pilot study data from a virtual reality simulation education intervention that compared virtual reality, augmented reality, serious gaming, and gamification.	The sample size and pending postintervention findings were stated as the limitations of the preliminary findings.
Boulet and Durning, 2019 [24]	United States	What we measure … and what we should measure in medical education	1	V	This paper focused on the validity of assessment scores and discusses the application of AI to automate the assessment process.	The authors recommended developing new competency assessment practices and highlighted the importance of the application of AI. They did not provide supporting evidence about AI's potential to eliminate the need for human ratings.
Conde et al, 2009 [25]	United States	Telehealth Innovations in Health Education and Training	1	V	This discussion paper indicated the potential of telehealth technologies for health education and training.	The authors recommended the development of AI applications for patient simulation and the integration of telehealth applications in health education, but the paper did not provide any evidence.
Kabassi et al, 2008 [26]	Greece	Specifying the personalization reasoning mechanism for an intelligent medical e-learning system on Atheromatosis: An empirical study	1	IV	The objective of this empirical study was to incorporate intelligent techniques in web-based medical education.	The authors described the specification of an intelligent medical learning system for atheromatosis that can interact with students. The design was based on the results of empirical data and the authors did not compare the e-learning system with traditional methods.
Klar and Bayer, 1990 [27]	Germany	Computer-assisted teaching and learning in medicine	1	IV	This article provided a comprehensive discussion of computer-assisted instruction systems and discussed expert systems' contribution to software for medical learning.	This paper was published before AI impacted multiple fields but the authors successfully envisioned how AI would transform decision making, simulation, and medical education.
Yang et al, 2019 [28]	Taiwan	An expert-led and artificial intelligence (AI) system-assisted tutoring course increase confidence of Chinese medical interns on suturing and ligature skills: prospective pilot study	1	IV	This paper examined the impact of an AI system tutoring course on clinical training.	This study compared regular, expert-led, and expert-led+AI groups and found an increased improvement in the expert-led+AI tutoring group. Authors recommended AI-assisted tutoring for novice medical interns.
Alonso-Silverio et al, 2018 [29]	Mexico	Development of a Laparoscopic Box Trainer Based on Open Source Hardware and Artificial Intelligence for Objective Assessment of Surgical Psychomotor Skills	1	IV	This study evaluated the effect of a laparoscopic trainer system that uses an AI algorithm.	The authors described the development of a low-cost intelligent simulator to improve laparoscopic skills and proposed the training as a validated training tool for surgical education programs.
Kolachalama and Garg, 2018 [30]	United States	Machine learning and medical education	2	V	This perspective article discussed the lack of student access to machine learning content and makes some suggestions to instructors.	This perspective paper only provided an outline and did not provide any evidence.
Park et al, 2019 [31]	Korea	What should medical students know about artificial intelligence in medicine?	2	IV	This short review emphasized the lack of direct access to machine learning education for clinicians and recommended the inclusion of focused content.	The review emphasized the need to identify correct information about AI.
Wartman and Combs, 2018 [32]	United States	Medical Education Must Move From the Information Age to the Age of Artificial Intelligence	2	V	This article discussed the need to develop new curricular components to teach the use of AI tools.	This commentary article summarized the authors' perspective and did not provide supporting evidence.
Wartman and Combs, 2019 [33]	United States	Reimagining Medical Education in the Age of AI	2	V	This paper indicated the need for a more sophisticated mathematical understanding of analytics.	The authors proposed a new curriculum that will include the skill sets required to use AI effectively.
Beregi, 2018 [34]	France	Artificial intelligence and medical imaging 2018: French Radiology Community white paper	2	IV	This review discussed current applications of AI in medical imaging and recommended AI education for radiology residents.	This position paper summarized AI principles, provided an update on research in the area of AI, and described radiologists' role in providing education about AI.
Tang et al, 2018 [35]	Canada	Canadian Association of Radiologists White Paper on Artificial Intelligence in Radiology	2	IV	This paper assessed the educational needs of radiologists and medical students, and provided recommendations.	The AI working group recommended the integration of health informatics and computer science courses to analyze the opportunities and challenges associated with new AI tools.
Masters, 2019 [36]	Oman	Artificial intelligence in medical education	2	V	This review highlighted the demand to learn how to work with AI systems and emphasized the need for AI training.	The authors identified new AI applications in medicine and recommended changes to medical curricula.
Chin-Yee and Upshur, 2017 [37]	Canada	Clinical judgement in the era of big data and predictive analytics	2	IV	This article explored different approaches to clinical judgment.	Authors indicated that data-driven and AI-based applications move medicine away from virtue-based approaches to clinical reasoning and recommended an integrative approach.
Santos et al, 2019 [38]	Germany	Medical students' attitude toward artificial intelligence: a multicenter survey	2	IV	This study investigated undergraduate medical students' attitudes toward AI.	The authors designed a survey to explore students' familiarity with AI concepts in radiology and concluded that they did not have an understanding of the basic technical principles underlying AI.
Paranjape et al, 2019 [39]	Netherlands	Introducing Artificial Intelligence Training in Medical Education	2	IV	This paper summarized the state of medical education and recommended a framework to include AI education.	This viewpoint paper suggested different AI-related content for different stages of medical education.

^a^Category 1: the use of AI applications in medical and health informatics education; Category 2: AI education.

^b^Evidence levels were as described by the Oxford Centre for Evidence-Based Medicine Levels of Evidence [40].

^c^AI: artificial intelligence.

^d^NLP: natural language processing.

**Figure 4:**
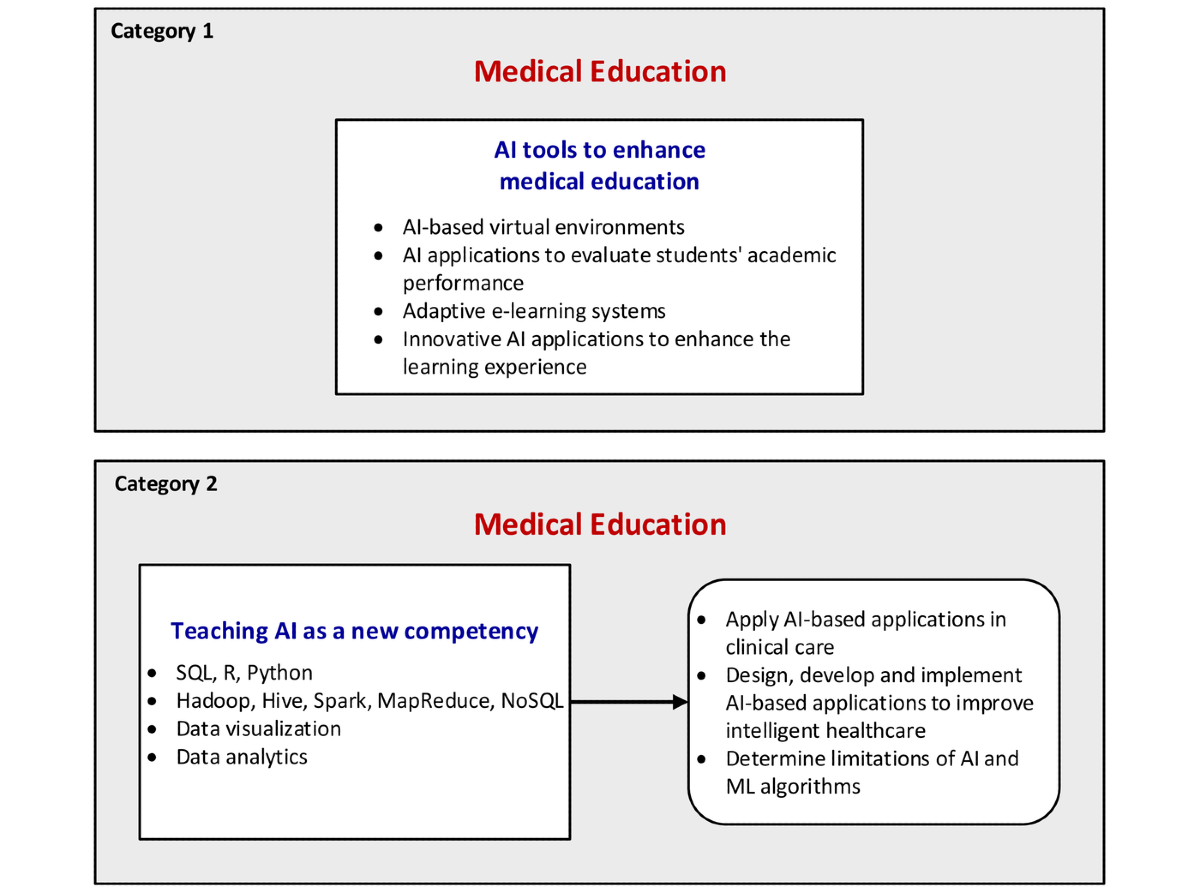
Classification of selected AI publications. AI: artificial intelligence; ML: machine learning.

Of 26 publications, 16 (61%) investigated the use of AI applications in medical education (Category 1) and 10 publications (39%) evaluated AI education in medicine (Category 2; Figure 4).

The first publications about the use of AI for medical applications were published in the early 1990s, and the capabilities of AI applications were restricted by technological limitations at that time. Klar and Bayer's paper [27], published in 1990, was one of the first publications about the application of AI, and they discussed the integration of expert knowledge into computer-assisted teaching in medicine. Lillehaug and Lajoie [16] proposed greater integration of AI in their 1998 paper, and they were among the first researchers who advocated for intelligent decision support systems and AI-based applications for medical education. Frize and Frasson [17] examined the role of decision support and intelligent tutoring systems in medical education, and recommended multidisciplinary studies in 2000.

Most Category 1 studies explored innovative applications designed to improve the learning experience. For example, Chan and Zary [15] evaluated existing AI applications in medicine, determined that the primary reason to use AI in medical education was to provide feedback, and identified that significant challenges included the assessment of effectiveness and management of technical difficulties. Another systematic review conducted by Chary et al [19] identified 30 articles that assessed the application of natural language processing (NLP) to medical education. NLP is a subfield of AI and refers to intelligent communication methods using natural languages. The study revealed the benefits of NLP training in residency education and recommended strategies for its application.

Simulation-based learning has evolved over the last decade, and the virtual environment has become essential for education. Our review identified multiple case studies about advanced virtual environments. For instance, Winkler-Schwartz et al [14] analyzed virtual reality simulators that use AI, and developed a checklist to assess studies using ML algorithms to evaluate technical skills. This study concluded that the checklist had the potential to decrease the knowledge gaps in the use of AI in surgical education. Similarly, Zhao et al [18] concluded that virtual patient systems and other distance education systems that used AI increased the efficiency of medical education. Another case study that focused on virtual patient simulators identified educational and technical challenges to enhancing the learning process with AI virtual reality applications [20]. The case study published by Bowyer et al [22] described the role of advanced virtual environments in surgical training. Moreover, Conde et al [25] recommended the use of AI applications for training and education when simulated human patients were not an option.

Augmented reality is another form of virtual reality, and this technology superimposes images on top of the video viewer. Sitterding et al [23] described the differences between virtual reality, augmented reality, serious gaming, and gamification, and shared the preliminary findings of their pilot study, which determined that the simulation experience was similar to a real-life environment.

In our research, we noted different innovative AI applications for special learning activities. In their 2017 article, Guimarães et al [21] reviewed current education models for anatomy education, and recommended the use of AI analytic tools to personalize the learning process. Boulet and Durning [24] discussed the application of AI to replace human ratings and assess medical education competencies. Another study that used augmented reality and AI algorithms evaluated a case study about the development of a laparoscopic box trainer to assess surgical psychomotor skills, and concluded the proposed system had potential benefits [29].

Incorporating intelligent techniques into an adaptive e-learning system was another research group's focus. Kabassi et al [26] designed a web-based educational system for medical education, incorporated intelligent algorithms to individualize the learning experience, and shared the findings. The authors recommended further studies with more participants. Yang and Shulruf [28] designed a pilot study to demonstrate the value of AI-assisted tutoring, and determined that this additional tutoring significantly enhanced the performance of medical students.

Our second research question involved determining the highest level of evidence of current research. The articles were classified according to the Oxford Centre for Evidence-Based Medicine (OCEBM) Levels of Evidence ranking scheme [40]. Of 26 articles, 18 (69%) were classified as Level IV evidence (case series), and 8 (31%) were classified as Level V evidence (expert opinions). The majority of the publications had Level IV and V evidence, which are considered poor reference standards. Hence, these findings emphasize the need to design new research studies.

Finally, our third research question was the following: what is the status of AI education in medicine and health informatics? In our study, 10 articles discussing AI education were identified. Since this topic is a relatively new area in medical and health informatics education, our findings were consistent with recent developments.

In their review, Paranjape et al [39] summarized multiple initiatives for AI in medical education, in which students worked with data experts and solved health care problems. The authors recommended familiarizing students with AI-based clinical applications, and introducing linear algebra, calculus, and probability during different stages of medical education. Similarly, Chin-Yee and Upshur [37] discussed the random error and biased data generated by AI and ML applications, and emphasized the effect of medical education for appraising clinical judgments.

A review conducted by Park et al [31] emphasized the importance of understanding AI to be able to validate the clinical accuracy of AI algorithms. Furthermore, multiple publications stated the need to move beyond traditional medical education, suggested a reform to align education with new practice requirements, and emphasized the role of academics and teachers in the development of appropriate AI application skills [32,33,36].

As discussed earlier, ML is an AI technique to process massive amounts of data and make predictions using computers. Kolachalama and Garg [30] proposed the integration of ML-related content in medical student, resident, and fellow education. The authors recommended the integration of real-world clinical examples into ML courses as well as practical guidelines for choosing the right tools.

The radiology community embraced AI and ML long before other medical specialties, and pioneered the usage of AI algorithms in advanced imaging applications. A multicenter survey of undergraduate medical students determined students' optimistic views about the implications of AI applications for radiology [38]. The French Radiology Community developed principles to regulate the use of AI tools, and recommended specific education to evaluate AI technologies [34]. Similarly, the Canadian Association of Radiologists recommended the integration of computer science, health informatics, and statistics training during residency education [35].

## Discussion

### Overview

The technological advancements in computer and software technologies; digitization of health care data; and methodological developments in information science, philosophy, mathematics, linguistics, and psychology disciplines accelerated medical research programs that focus on ML and commercialization. Maturation of AI technologies changed the roles of clinicians, and novel decision-making processes in medical settings and innovative AI-based protocols have the potential to provide diagnostic and treatment decisions by analyzing complex data sets [41].

Over the last decade, many AI researchers have concentrated on developing a proof of concept system for clinicians and patients. There is an increase in the number of studies that evaluate the effectiveness of intelligent reasoning. Intelligent monitoring technologies require new algorithms to detect anomalies, predict patterns, and make decisions.

A recent independent report prepared for the UK Secretary of State for Health and Social Care explored how the health care workforce could be prepared to use digital technology. The report emphasized the skills gap in the workforce, and made some recommendations about the integration of digital health care technologies, AI, and data analytics in undergraduate curricula [42,43].

Clinicians should have a realistic view of AI, and become familiar with the right tasks for AI in health care. Formal training for medical and health informatics students should enable them to develop AI algorithms, use AI technologies in a competent manner, and keep bias out of AI tools. Any new AI technology might encounter something new for which it has no experience, and therefore the physician should be able to assess problematic decisions and take necessary precautions when needed. Recent discussions about the need for medical education reform emphasize the shortcomings of the current model of education [44]. Overreliance on ML and AI technologies might have unintended severe adverse consequences, such as failure to recognize invalid test results [45].

While machine learning and AI algorithms are able to handle high-dimensional data classification problems and medical image interpretation, their success rates in risk prediction and diagnosis are lower. Consequently, there is a need to determine the most appropriate application areas for AI in health care [46].

There are different ways to implement AI in clinical practice, and clinicians and health informaticians need formal training to use the right approaches. Health informaticians and physician champions who design and develop AI-based protocols need to have a good understanding of complex algorithms, methodologies for data quality assessment, probabilistic forecasting, and comparative model assessment to work with engineers and develop reliable AI applications. Moreover, clinicians who use AI applications should become familiar with potential challenges.

AI-based relational time pattern analysis replaced simple threshold-based diagnostic rules. Current medical education and health informatics curricula still do not provide the ability to understand AI communications and necessary skill sets to develop AI systems that can detect and analyze relational time patterns [41].

The ability to interpret AI algorithms' mistakes and formulate the best strategies to correct these applications requires specialized training. Consequently, medical and health informatics education must emphasize algorithm-based platforms, and include relevant data analytics and AI topics in their curricula. Moreover, computer science and health informatics programs should consist of health care–focused digital skills training.

To the best of our knowledge, no previous research has investigated the use of AI tools to enhance the learning experience and AI education of medical and health informatics students. The main findings of this systematic review are as follows: (1) Although there are several recommendations on the integration of AI into medical and health informatics curricula and some academic institutions implemented experimental training programs, AI and ML education are not a part of traditional medical and health informatics curricula yet. (2) Current medical education and health informatics accreditation standards do not require AI training, and AI competencies have not been determined. (3) Using the OCEBM Levels of Evidence classification table, the majority of studies were classified as Level IV and V, which indicates poor reference standards.

### Limitations

Several efforts were made to design an optimal systematic review process; however, there were still many limitations. It is probable that some studies might not be listed in the peer-reviewed academic literature databases or might be published in a non-English language. Although this is a systematic review of the field, AI is a new technical discipline, particularly in medicine, and therefore the number of articles that met the inclusion criteria was limited.

### Future Directions

Overall, the selected publications did not provide specific details about different jobs' requirements and curriculum needs. The emergence of intelligent systems in health care requires new learning modalities. Even though several organizations, agencies, and work groups such as the International Medical Informatics Association [47], the Commission on Accreditation for Health Informatics and Information Management Education [48,49], the Health Informatics Society of Australia [50], the TIGER (Technology Informatics Guiding Education Reform) Initiative [51], and the Association of American Medical Colleges [52] published skill recommendations for health informatics curricula, they have not determined specific skill sets for AI education. Furthermore, a recent study evaluated health informatics students' skills in developing AI apps and emphasized the need to develop new competencies [53].

**Figure 5:**
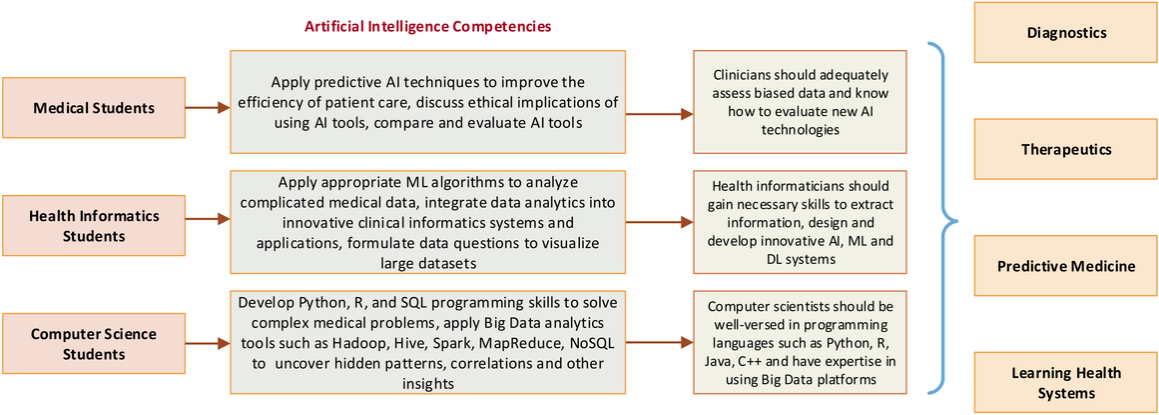
Proposed framework for specialized AI training for different professional fields. AI: artificial intelligence; DL: deep learning; ML: machine learning.

A specialized AI education framework for various professional fields would be useful; using the results of this systematic review, we propose a framework for specialized AI training for different domains (Figure 5). Medical students need to become familiar with clinical AI applications and predictive modeling techniques to assess biased data and evaluate innovative AI technologies. Health informatics students should become familiar with the application of appropriate ML algorithms and development of innovative clinical informatics systems. Furthermore, they should gain the hands-on skills required to extract data, manage large data sets to perform sophisticated data analytics, and develop innovative AI systems. Computer science students need specialized skill sets to work with data scientists and should become familiar with Python, R, and SQL programming languages, and data analytics tools (Figure 5).

## Abbreviations

AI: artificial intelligence
CINAHL: Cumulative Index to Nursing and Allied Health Literature
DL: deep learning
IEEE: Institute of Electrical and Electronics Engineers
ML: machine learning
NLP: natural language processing
OCEBM: Oxford Centre for Evidence-Based Medicine
PRISMA-P: Preferred Reporting Items for Systematic Reviews and Meta-Analysis Protocols
TIGER: Technology Informatics Guiding Education Reform
